# Assessing a Smartphone App (AICaries) That Uses Artificial Intelligence to Detect Dental Caries in Children and Provides Interactive Oral Health Education: Protocol for a Design and Usability Testing Study

**DOI:** 10.2196/32921

**Published:** 2021-10-22

**Authors:** Jin Xiao, Jiebo Luo, Oriana Ly-Mapes, Tong Tong Wu, Timothy Dye, Nisreen Al Jallad, Peirong Hao, Jinlong Ruan, Sherita Bullock, Kevin Fiscella

**Affiliations:** 1 Eastman Institute for Oral Health University of Rochester Rochester, NY United States; 2 Computer Science University of Rochester Rochester, NY United States; 3 Department of Biostatistics and Computational Biology University of Rochester Medical Center Rochester, NY United States; 4 Department of Obstetrics and Gynecology University of Rochester Medical Center Rochester, NY United States; 5 Healthy Baby Network Rochester, NY United States; 6 Department of Family Medicine University of Rochester Medical Center Rochester, NY United States

**Keywords:** artificial intelligence, smartphone app, mDentistry, dental caries, underserved population, mobile dentistry

## Abstract

**Background:**

Early childhood caries (ECC) is the most common chronic childhood disease, with nearly 1.8 billion new cases per year worldwide. ECC afflicts approximately 55% of low-income and minority US preschool children, resulting in harmful short- and long-term effects on health and quality of life. Clinical evidence shows that caries is reversible if detected and addressed in its early stages. However, many low-income US children often have poor access to pediatric dental services. In this underserved group, dental caries is often diagnosed at a late stage when extensive restorative treatment is needed. With more than 85% of lower-income Americans owning a smartphone, mobile health tools such as smartphone apps hold promise in achieving patient-driven early detection and risk control of ECC.

**Objective:**

This study aims to use a community-based participatory research strategy to refine and test the usability of an artificial intelligence–powered smartphone app, AICaries, to be used by children’s parents/caregivers for dental caries detection in their children.

**Methods:**

Our previous work has led to the prototype of AICaries, which offers artificial intelligence–powered caries detection using photos of children’s teeth taken by the parents’ smartphones, interactive caries risk assessment, and personalized education on reducing children’s ECC risk. This AICaries study will use a two-step qualitative study design to assess the feedback and usability of the app component and app flow, and whether parents can take photos of children’s teeth on their own. Specifically, in step 1, we will conduct individual usability tests among 10 pairs of end users (parents with young children) to facilitate app module modification and fine-tuning using think aloud and instant data analysis strategies. In step 2, we will conduct unmoderated field testing for app feasibility and acceptability among 32 pairs of parents with their young children to assess the usability and acceptability of AICaries, including assessing the number/quality of teeth images taken by the parents for their children and parents’ satisfaction.

**Results:**

The study is funded by the National Institute of Dental and Craniofacial Research, United States. This study received institutional review board approval and launched in August 2021. Data collection and analysis are expected to conclude by March 2022 and June 2022, respectively.

**Conclusions:**

Using AICaries, parents can use their regular smartphones to take photos of their children’s teeth and detect ECC aided by AICaries so that they can actively seek treatment for their children at an early and reversible stage of ECC. Using AICaries, parents can also obtain essential knowledge on reducing their children’s caries risk. Data from this study will support a future clinical trial that evaluates the real-world impact of using this smartphone app on early detection and prevention of ECC among low-income children.

**International Registered Report Identifier (IRRID):**

PRR1-10.2196/32921

## Introduction

Early childhood caries (ECC) is by far the most common chronic childhood disease, with nearly 1.8 billion new cases per year worldwide [1-3]. In the United States, it afflicts approximately 37% of all children aged 2-5 years but disproportionately affects up to 55% of low-income and minority children. Untreated ECC often leads to higher risk of caries lesions in permanent teeth, diminished oral health–related quality of life, hospitalizations and emergency room visits due to systemic infection, and even death [4,5]. Hence, more innovative/effective preventive and treatment strategies are needed, particularly early detection of ECC.

The current biomedical approach to control the ECC pandemic has had limited success. Primarily, this approach focuses on individual-level restorative procedures rather than populationwide preventive strategies. Dental caries is localized destruction of dental hard tissues (enamel and dentin) by acidic by-products from the microbial fermentation of carbohydrates. In the early (subclinical) stages, such as white spot lesions on the tooth enamel surface, caries can be reversed. Many US preschool children from low-income families, however, often have poor access to pediatric dental services; limited dental access leave the underserved children in positions where dental caries is often diagnosed in later stages, thus requiring more extensive restorative treatments. Moreover, ECC is a multifactorial disease with host, microorganisms, diet, and oral hygiene practices as the factors that determine the risks [6-9]. Children’s parents/caregivers need to be engaged around these risk factors and acquire skills to self-manage risk to reduce children’s risk for ECC.

To combat this ECC pandemic and overcome the barriers of lacking dental access among underserved children and lacking self-management awareness of these children’s caregivers, our long-term goal is to develop a first-of-its-kind artificial intelligence (AI)–powered smartphone app to be used by children’s parents, which offers patient-centered caries detection and caries risk management.

Smartphone apps have been successfully applied in managing individual behaviors and health conditions [10] such as smoking cessation, weight loss, medication adherence, and Parkinson disease progression monitoring [10-12]. With 77% of all age American individuals [13] and more than 85% of lower-income mid-age American individuals [14,15] owning a smartphone, a smartphone app presents as a suitable and innovative way of providing oral health interventions. Recently, mobile dentistry has been brought up by researchers to promote oral health care at a broad population base [16,17]; however, current oral health smartphone apps are limited in scope and audience. First, compared to the large amount of available medical health apps in the commercial app store, the number of apps that are oral health focused is minimal. Second, most of the available apps are designed for improving the efficiency of tooth brushing or helping users understand oral disease types and manifestation. There is no technology, much less any app, that can be used by parents for early detection of caries in their children. Furthermore, using AI to aid imaging recognition has been applied to improve disease diagnosis in many medical fields including oncology, ophthalmology, radiology, etc [18-21]. However, modern dentistry has not used AI imaging technology for caries detection. To our knowledge, AICaries will be the first app using this technology in dentistry.

In summary, a patient-friendly smartphone app coupled with AI-powered caries detection holds promise in facilitating early clinical confirmation and treatment of ECC. Led by experts in AI imaging recognition, oral health, and mobile health (mHealth), this AICaries study will address the gap in research and clinical practice for ECC from the angle of disease early detection and self-management using mHealth tools.

## Methods

### Aims

The purpose of the study is to use a community-based participatory research strategy to refine and test the usability of an AI-powered smartphone app, AICaries, to be used by children’s parents/caregivers for dental caries detection in their children.

### Overall Study Design

#### Phase I: Refine the AICaries App

**Figure 1:**
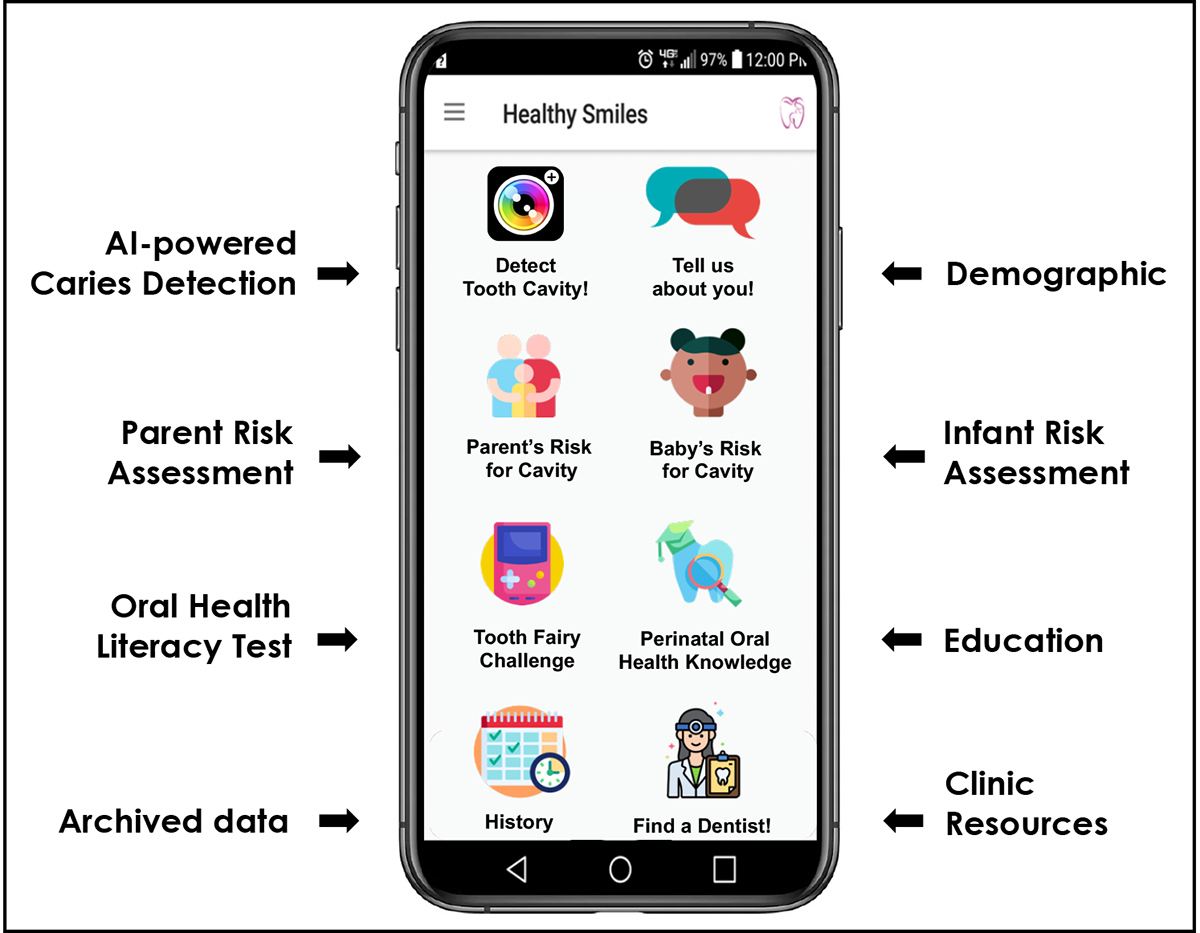
Components of AICaries smartphone app. The AICaries app prototype houses the AICaries tool for caries detection and other developed app components including maternal and child’s caries risk assessment, and education and clinic resources. AI: artificial intelligence.

We used an integrate, design, assess, and share framework [22] and developed a smartphone app prototype (AICaries) to be used by children’s parents. The app shell includes the content shown in Figure 1. Although some components of the app are to be used specifically by mothers, the components that are related to the children could be used by both mothers and fathers of the children.

- AI-powered caries detection algorithm: our team has archived a data set with more than 100,000 high-quality intraoral photos including front and posterior teeth. Using a semiauto dental caries annotation software developed in house, trained and calibrated dentists have annotated approximately 40,000 individual tooth photos for dental caries using the International Caries Diagnosis System [23,24] visual diagnostic criteria as the reference for caries detection and severity scoring. These annotated tooth images were used for developing the AI-powered caries detection algorithm. The performance of the AICaries caries detection algorithm will be assessed in a separate study.
- Maternal risk assessment and child risk assessment: an interactive caries risk assessment tool for mothers and young children. We modified the elements in the American Dental Association (ADA) Caries Risk Assessment system for less health-literate individuals. Through these assessments, mothers can visualize how daily oral hygiene behavior and diet could impact their and their children’s caries risk.
- Validated perinatal oral health literacy index: to measure mother’s oral health literacy [25,26]
- Maternal and children’s oral health education resources: we assembled a series of informative educational materials that provide appropriately timed information specific to pregnant women’s oral health importance, children’s tooth development, children’s oral hygiene, and diet recommendations.
- List of available dental clinics that accept dental insurance for low-income groups (eg, Medicaid): built upon the list developed by the University of Rochester Eastman Institute for Oral Health (EIOH) and our community partner Healthy Baby Network via a New York State Department of Health grant “MICHC Oral Health Manual and Toolkit”

The AICaries app prototype houses the AICaries tool for caries detection and other developed app components including maternal and child’s caries risk assessment, and education and clinic resources.

#### Phase II: Usability Testing

This AICaries study will use the qualitative study design to assess the feedback and usability of the app component, app flow, and whether parents can take photo of children’s teeth on their own. The proposed human study will not assess the AI performance, which will be assessed in a separate study.

**Figure 2:**
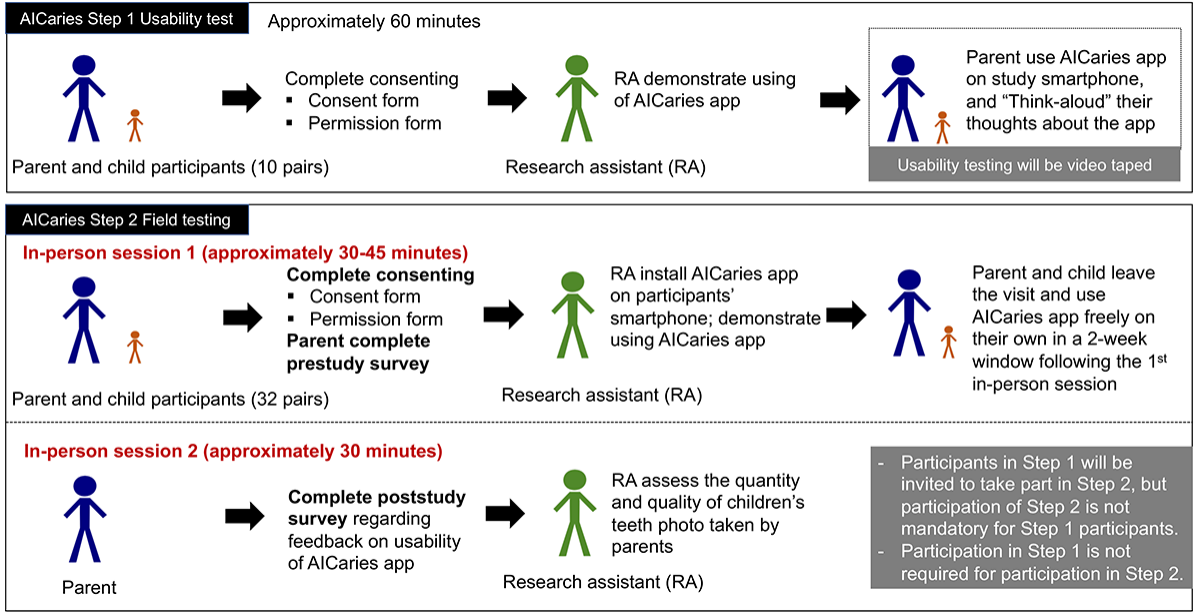
AICaries usability test study flow.

Specifically, we will conduct iterative moderated usability testing and refinement for the AICaries app that was developed and will be refined by our study team. Briefly, we will conduct the study in 2 steps, detailed in Figure 2.

- Step 1 AICaries usability test: we will conduct individual usability tests among 10 pairs of end users (parents with young children: 10 parents and 10 children) to facilitate app module modification and fine-tuning using think-aloud and instant data analysis strategies. Only one study session was designed.
- Step 2 AICaries field testing: we will conduct unmoderated field testing for app feasibility and acceptability. Field testing will be conducted in the real world among 32 pairs of parents with their young children (a total of 64 participants: 32 parents and 32 children younger than 5 years) to assess the usability and acceptability of AICaries, including assessing the number/quality of teeth images taken by the parents for their children and parents’ satisfaction. Two study sessions are designed (detailed in the section Study Procedures).

### Participants

A total of up to 84 participants, 42 parents and 42 children younger than 5 years of age will be enrolled in the study. The study population will consist primarily of economically and socially disadvantaged parents 18 years and older (mainly mothers) and their young children (<5 years of age). We expect the participants to be 40% White, 45% Black or African American, 5% Asian, and 10% other race; we expect the composition of ethnical groups among the study sample is 80% non-Hispanic and 20% Hispanic. Since mothers are the primary caregiver for children, the ratio between female and male of the parent participants is expected to be 4:1, with 80% mothers and 20% fathers. Both male and female children will be recruited. Detailed inclusion and exclusion criteria are shown in Textbox 1.

**Inclusion and exclusion criteria.**

**Parents who have young children**

- Inclusion criteria
  Provide signed and dated informed consent
  Have an Android smartphone that could be installed with the AICaries app
  Willing to comply with all study procedures and be available for the duration of the study
  Female or male, 18 years or older
  Have at least 1 child aged between 1-5 years
  Eligible for state-supported Medicaid type of insurance (eg, Medicaid, Blue Choice, MVP Option, Fidelis Medicaid; note, we are using the insurance eligibility to select low-income participants)
  English speaking
- Exclusion criteria
  Participants who have decisional impairment and are incapable of making an informed decision about their participation in the study

**Preschool children**

- Inclusion criteria
  Female or male, aged between 1-5 years
  Eligible for state-supported Medicaid type of insurance (eg, Medicaid, Blue Choice, MVP Option, Fidelis Medicaid)
- Exclusion criteria
  Orofacial deformity (cleft lip, cleft palate, oral-pharyngeal mass)
  Down syndrome or other developmental disabilities

### Recruitment

We will conduct face-to-face recruitment at the following two sites. The characteristics of these clinics make them suitable for the recruitment in the proposed study.

1. University of Rochester Medical Center (URMC)–EIOH Perinatal Dental Clinic: the URMC-EIOH Perinatal dental clinic was founded in March 2018 and is dedicated to serving socioeconomically disadvantaged pregnant women, women post partum, and young children. Since its founding, URMC-EIOH Perinatal Dental Clinic has provided dental care to more than 300 mothers and their children. A majority of the patients at the EIOH Perinatal Dental Clinic are eligible for state-supported Medicaid type of insurance, which is one of the eligibility requirements of the study.
2. URMC, Highland Family Medicine (HFM): URMC-HFM is one of the largest family medicine residency teaching practices in Monroe County, New York. HFM provides comprehensive prenatal care to the mothers and care to the baby from birth through childhood. Medical records showed that more than 600 children younger than 5 years (44% African American, 39% Caucasian, 7% Asian, and 10% other) are seen at HFM each year.

### Retention Strategies

The AICaries step 2 field testing involves 2 in-person study sessions. The research assistant (RA) will phone or text to remind participants about the upcoming study visits approximately 3 days prior to the session. We will try to accommodate their social, economic, or physical barriers to coming for a study session, such as providing transportation and parking assistance.

### Study Procedures

#### Step 1 AICaries Usability Test

We will conduct 10 in-person, one-on-one, 60-minute usability tests of the smartphone to facilitate app module modification and fine-tuning using think-aloud [27] and instant data analysis strategies [27].

- The parent will be asked to bring one child with them to the study site (URMC-EIOH), involving a quiet room equipped with Wi-Fi and videotaping, staffed by two trained RAs.
- Following informed consent, the RA who is moderating will ask the parent to complete a brief survey (part 1 of Multimedia Appendix 1) that includes the age of the parent and child, parent’s sex, educational level, frequency of use of smartphone apps, and use of phone for photographs. The second RA will monitor the child.
- Prior to the testing, one RA will briefly demonstrate how to use the app and think-aloud strategy to express their feedback while testing the app.
- The parent will then use the app while thinking aloud throughout the entire process.
- The key usability tasks include navigation of the app interface; accessing and completing the ADA caries risk assessment; and, notably, taking usable (diagnostic) photographs of their child’s front teeth using the photo-taking interface.
- The RA will provide encouragement and affirmation throughout, and only offer assistance if the parent hits a clear road block. The RA will provide facilitating statements (eg, “I noticed you did X. Can you explain why?”) or echoing comments. At the end of the session, the parent will be asked about suggestions for improvement.
- Each session will be videotaped. The recording will start with participants’ verbal permission. We will inform the participants when the recording stops. The videotape will be reviewed by the study team immediately after the session using instant data analysis [27] with attention to photo image quality following assessment criteria in parts 3 and 4 in Multimedia Appendix 1. Each user challenge will be ranked as critical (required assistance to proceed), moderate (major delay or frustration), or cosmetic (minor) and annotated to the exact interface/task. Based on rankings, the team will suggest changes in the instructional video, app design, and study procedures. The changes will be incorporated into subsequent testing sessions.

#### Step 2 AICaries Field Testing

The unmoderated field testing allows the end users to interact with the AICaries app in their natural environment. We will enroll 32 pairs of parents and their young children, a total of 64 eligible participants, for step 2 of the AICaries study. There are two in-person study sessions.

1. At the first in-person session, approximately 30 to 45 minutes, following informed consent, the participants will complete a baseline questionnaire (part 1 of Multimedia Appendix 1. The RA will then install the AICaries app on participants’ smartphone. After the participants complete the first study visit, over the course of the next 2 weeks, they will test the app and take photo images of their child’s front teeth only (no facial features) and send them to the study team via SMS text messages. If the participating parent fails to send their child’s teeth photo to the study team by the end of 2 weeks, our RA will call or text participating parents to remind them about the study activities.
2. The second in-person study session will take place after the 2-week app testing; the participants will come to the study site, complete a brief in-person survey (part 2 of Multimedia Appendix 1) of Likert items that address ease-of-use of the instructional support quality, ease-of-learning to take correct photographs, and overall satisfaction. App use metrics will include quantity and quality of children’s teeth images taken by the parents. The participants will also be interviewed about their experience of using AICaries based on a semistructured interview guide (part 5 of Multimedia Appendix 1). Interview sessions will be audio recorded, transcribed, and analyzed qualitatively for thematic content. Additionally, the RA will debrief with the participant and elicit suggestions for improvement.

### Statistical Analysis

#### Sample Size Considerations

##### Step 1 AICaries Usability Test

We expect to reach data saturation [28] (no new suggestions are proposed) after conducting 10 individual usability tests. The sample size is determined based on previous published studies, where between 6 to 11 usability tests were conducted to assess smartphone app usability [29,30].

##### Step 2 AICaries Field Testing

A sample size of 28 produces a two-sided 95% CI with a width equal to 0.3 when 80% of parents produce usable tooth images for their children using AICaries. Considering a dropout rate of 10%, we plan to recruit 32 parents and their children.

#### Data Analysis

##### Qualitative Data

The transcribed data for the step 1 AICaries usability test and step 2 AICaries field testing will be coded with predetermined open codes. Thematic content will be further analyzed using categorizing and contextualizing strategies to understand the needed improvement for the AICaries app.

##### System Usability Scale

For the step 2 AICaries field testing, the System Usability Scale (SUS) will be used to assess the acceptance of the AICaries (part 2 of Multimedia Appendix 1). The SUS instrument [31-33] is widely adopted in business and technology industries and mHealth fields to measure and quantify the perception of product and service usability. A SUS score above 68 indicates above-average usability; a score above 80.3 indicates excellent usability of the AICaries app. Baseline information of the participants that are collected, such as previous use of smartphone apps or taking part in the step 1 study, which might make participants more familiar with the app, will be taken into consideration during the usability analysis. Additional quantitative data that will be analyzed are app use metrics that will include which screens opened, time between screens, and numbers/quality (whether photos could be used for clinical diagnosis for dental caries) of images taken (part 5 of Multimedia Appendix 1).

## Results

The study has been peer reviewed and funded by the National Institute of Dental and Craniofacial Research, United States. The research ethics application has been reviewed and approved by the University of Rochester Research Subject Review Board (IRB STUDY00005772, 00003953, 00005949). The AICaries smartphone app usability was launched on August 6, 2021. Data collection and analysis are expected to conclude by March 2022 and June 2022, respectively.

## Discussion

### Study Innovation

National surveys in the United States have shown that low-income and minority children not only are disproportionately affected by ECC but also have limited access to oral health care [34,35]. The percentage of US children aged 0-6 years with at least one dental visit is much lower among families whose income are lower than the federal poverty line [34,35]. One way to address this health system dilemma is to make the oral disease screening service accessible to individual patients regardless of their socioeconomic condition, such as via mHealth tools. Currently, patients can monitor their blood pressure via home use blood pressure devices; patients can monitor their blood glucose via home use glucose meter; patients can even monitor their heart rate and rhythm via a smartwatch. In contrast, when monitoring oral health, other than visiting dental professionals, patients have no way to monitor their oral diseases using a personal device. The AICaries smartphone app will be a first-of-its-kind patient-centered caries early detection and screening tool, and a useful resource for caries risk assessment and oral health education.

### Implications

Using AICaries, parents can use their regular smartphones to take photos of their children’s teeth and detect ECC, aided by AICaries, so that they can actively seek treatment for their children at an early and reversible stage of ECC. Using AICaries, parents can also obtain essential knowledge on reducing their children’s caries risk. Data from this study will support a future clinical trial that will evaluate the real-world impact of using this innovative smartphone app on early detection and prevention of ECC among low-income children.

## Appendix

Multimedia Appendix 1 Data collection and assessment forms.

Multimedia Appendix 2 National Institutes of Health grant peer-review summary.

## Abbreviations

ADA: American Dental Association
AI: artificial intelligence
ECC: early childhood caries
EIOH: Eastman Institute for Oral Health
HFM: Highland Family Medicine
mHealth: mobile health
RA: research assistant
SUS: System Usability Scale
URMC: University of Rochester Medical Center

## References

[1] Dye BA, Li X, Thorton-Evans G (2012). Oral health disparities as determined by selected healthy people 2020 oral health objectives for the United States, 2009-2010. NCHS Data Brief.

[2] Dye BA, Tan S, Smith V, Lewis BG, Barker LK, Thornton-Evans G, Eke PI, Beltrán-Aguilar ED, Horowitz AM, Li C (2007). Trends in oral health status: United States, 1988-1994 and 1999-2004. Vital Health Stat 11.

[3] GBD 2016 Disease and Injury Incidence and Prevalence Collaborators (2017). Global, regional, and national incidence, prevalence, and years lived with disability for 328 diseases and injuries for 195 countries, 1990-2016: a systematic analysis for the Global Burden of Disease Study 2016. Lancet.

[4] American Academy of Pediatric Dentistry Council on Clinical Affairs (2005). Policy on early childhood caries (ECC): unique challenges and treatment options. Pediatr Dent.

[5] Casamassimo PS, Thikkurissy S, Edelstein BL, Maiorini E (2009). Beyond the dmft: the human and economic cost of early childhood caries. J Am Dent Assoc.

[6] Meng Y, Wu T, Billings R, Kopycka-Kedzierawski DT, Xiao J (2019). Human genes influence the interaction between Streptococcus mutans and host caries susceptibility: a genome-wide association study in children with primary dentition. Int J Oral Sci.

[7] Mack H, Basabe JV, Brossmer R (1988). 2-Acetamido-2-deoxy-D-gluco- and -D-manno-furanose: a simple preparation of 2-acetamido-2-deoxy-D-mannose. Carbohydr Res.

[8] Xiao J, Huang X, Alkhers N, Alzamil H, Alzoubi S, Wu TT, Castillo DA, Campbell F, Davis J, Herzog K, Billings R, Kopycka-Kedzierawski DT, Hajishengallis E, Koo H (2018). Candida albicans and early childhood caries: a systematic review and meta-analysis. Caries Res.

[9] Wu TT, Xiao J, Sohn MB, Fiscella KA, Gilbert C, Grier A, Gill AL, Gill SR (2021). Machine learning approach identified multi-platform factors for caries prediction in child-mother dyads. Front Cell Infect Microbiol.

[10] Ernsting C, Dombrowski SU, Oedekoven M, O Sullivan JL, Kanzler M, Kuhlmey A, Gellert P (2017). Using smartphones and health apps to change and manage health behaviors: a population-based survey. J Med Internet Res.

[11] Wang J, Wang Y, Wei C, Yao NA, Yuan A, Shan Y, Yuan C (2014). Smartphone interventions for long-term health management of chronic diseases: an integrative review. Telemed J E Health.

[12] Dorsey ER, Glidden AM, Holloway MR, Birbeck GL, Schwamm LH (2018). Teleneurology and mobile technologies: the future of neurological care. Nat Rev Neurol.

[13] (2021). Mobile fact sheet. Pew Research Center.

[14] Chowdhry A (2018). Lower-income Americans are becoming increasingly dependent on smartphones, says study. Forbes.

[15] Vangeepuram N, Mayer V, Fei K, Hanlen-Rosado E, Andrade C, Wright S, Horowitz C (2018). Smartphone ownership and perspectives on health apps among a vulnerable population in East Harlem, New York. Mhealth.

[16] Xiao J, Fiscella KA, Meyerowitz C (2021). mDentistry: a powerful tool to improve oral health of a broad population in the digital era. J Am Dent Assoc.

[17] Wang L, Ren J, Fiscella KA, Bullock S, Sanders MR, Loomis EL, Eliav E, Mendoza M, Cacciato R, Thomas M, Kopycka-Kedzierawski DT, Billings RJ, Xiao J (2020). Interprofessional collaboration and smartphone use as promising strategies to improve prenatal oral health care utilization among US underserved women: results from a qualitative study. BMC Oral Health.

[18] Azuaje F (2019). Artificial intelligence for precision oncology: beyond patient stratification. NPJ Precis Oncol.

[19] Hosny A, Parmar C, Quackenbush J, Schwartz LH, Aerts HJWL (2018). Artificial intelligence in radiology. Nat Rev Cancer.

[20] Robertson S, Azizpour H, Smith K, Hartman J (2018). Digital image analysis in breast pathology-from image processing techniques to artificial intelligence. Transl Res.

[21] Lu W, Tong Y, Yu Y, Xing Y, Chen C, Shen Y (2018). Applications of artificial intelligence in ophthalmology: general overview. J Ophthalmol.

[22] Mummah SA, Robinson TN, King AC, Gardner CD, Sutton S (2016). IDEAS (Integrate, Design, Assess, and Share): A Framework and Toolkit of Strategies for the Development of More Effective Digital Interventions to Change Health Behavior. J Med Internet Res.

[23] Ginnis J, Ferreira Zandoná AG, Slade GD, Cantrell J, Antonio ME, Pahel BT, Meyer BD, Shrestha P, Simancas-Pallares MA, Joshi AR, Divaris K (2019). Measurement of early childhood oral health for research purposes: dental caries experience and developmental defects of the enamel in the primary dentition. Methods Mol Biol.

[24] Shoaib L, Deery C, Ricketts DNJ, Nugent ZJ (2009). Validity and reproducibility of ICDAS II in primary teeth. Caries Res.

[25] Hom JM, Lee JY, Divaris K, Baker AD, Vann WF (2012). Oral health literacy and knowledge among patients who are pregnant for the first time. J Am Dent Assoc.

[26] Dickson-Swift V, Kenny A, Farmer J, Gussy M, Larkins S (2014). Measuring oral health literacy: a scoping review of existing tools. BMC Oral Health.

[27] Joe J, Chaudhuri S, Le T, Thompson H, Demiris G (2015). The use of think-aloud and instant data analysis in evaluation research: exemplar and lessons learned. J Biomed Inform.

[28] Morse JM (2016). Determining sample size. Qual Health Res.

[29] Reeder B, Drake C, Ozkaynak M, Wald HL (2019). Usability testing of a mobile clinical decision support app for urinary tract infection diagnosis in nursing homes. J Gerontol Nurs.

[30] Simons D, De Bourdeaudhuij I, Clarys P, De Cocker K, Vandelanotte C, Deforche B (2018). A smartphone app to promote an active lifestyle in lower-educated working young adults: development, usability, acceptability, and feasibility study. JMIR Mhealth Uhealth.

[31] Lopes JP, Dias TMR, Carvalho DBF, Oliveira JFD, Cavalcante RB, Oliveira VCD (2019). Evaluation of digital vaccine card in nursing practice in vaccination room. Rev Lat Am Enfermagem.

[32] Friesen EL (2017). Measuring AT usability with the modified System Usability Scale (SUS). Stud Health Technol Inform.

[33] Pillalamarri SS, Huyett LM, Abdel-Malek A (2018). Novel bluetooth-enabled tubeless insulin pump: a user experience design approach for a connected digital diabetes management platform. J Diabetes Sci Technol.

[34] Kopycka-Kedzierawski DT, McLaren SW, Billings RJ (2018). Advancement of teledentistry at the University Of Rochester's Eastman Institute For Oral Health. Health Aff (Millwood).

[35] Edelstein BL (2002). Disparities in oral health and access to care: findings of national surveys. Ambul Pediatr.

