# Supplementary material for: Assessing a Smartphone App (AICaries) That Uses Artificial Intelligence to Detect Dental Caries in Children and Provides Interactive Oral Health Education: Protocol for a Design and Usability Testing Study
Source: JMIR Res Protoc. 2021 Oct 22;10(10):e32921. doi: 10.2196/32921 (PMC8571694; doi:10.2196/32921)
Supplement: Multimedia Appendix 1 [file resprot_v10i10e32921_app1.docx]

**Appendix 1: AICaries Baseline questionnaire**

**(To be used for Step 1 Usability study and Step 2 Field testing study)**

Please answer the following questions, thank you!

***Part 1: Social-demographic background***

**Name** ________________________ **Birth date**________________ **Sex:** F M Others

**Race:** American Indian/Alaska Native Asian Native Hawaiian or Pacific Islander Black or African American

Caucasian More than one race Unknown or unreported

**Ethnicity:** Hispanic Non-Hispanic

If minor, parents’ names _____________________________ Relation to the study subject __________________________

Home phone _____________ Cell phone _______________ Email address _____________________________________

Mailing address ____________________________________ City ________________ State ________ Zip ___________

1. What is your current work status?

_1_ I am currently employed

_2_ I am currently unemployed

2. If currently employed, what is your most recent occupation?

3. What is the highest grade you completed?

_1_ Middle school

_2_ High school

_3_ More than High School

_4_ College level

_5_ Postgraduate level

4. What is your marital status?

_1_ Married

_2_ Single

_3_ Separated

_4_ Divorced

_5_ Widowed

_6_ Other

***5. Does your child attend daycare?***

_1_ No

_2_ Yes, part time

_3_ Yes, full time

***Part 2: Electronic device use***

6. Do you use medical care related Apps on your phone (e.g., Mychart, Healow, SimplePractice)?

A. Yes

B. No

7. Do you use dental care related Apps on your phone?

A. Yes (Please specify________)

B. No

C. I am not sure what Apps you are referring to

8. Have you tried to use a cell phone to take photos of your teeth or mouth, or your child’s teeth?

A. Yes, many times

B. Yes, a few times

C. Never

**Appendix 2: AICaries post-study questionnaire**

**(To be used for Step 2 field testing study in-person session 2)**

**Instruction:** For each of the following statements, mark one box that best describes your thoughts on using AICaries app.

| No | Question description | Strongly disagree |  |  |  | Strongly agree | Score |
| --- | --- | --- | --- | --- | --- | --- | --- |
| 1 | I think that I would like to use the AICaries app frequently. |  |  |  |  |  |  |
|  | *Scoring algorism (point)* | 0 | 1 | 2 | 3 | 4 |  |
| 2 | I found the AICaries app unnecessarily complex. |  |  |  |  |  |  |
|  | *Scoring algorism (point)* | 4 | 3 | 2 | 1 | 0 |  |
| 3 | I thought the AICaries app was easy to use. |  |  |  |  |  |  |
|  | *Scoring algorism (point)* | 0 | 1 | 2 | 3 | 4 |  |
| 4 | I think that I would need the support of a technical person to be able to use the AICaries app. |  |  |  |  |  |  |
|  | *Scoring algorism (point)* | 4 | 3 | 2 | 1 | 0 |  |
| 5 | I found the various components in the AICaries app were well integrated. |  |  |  |  |  |  |
|  | *Scoring algorism (point)* | 0 | 1 | 2 | 3 | 4 |  |
| 6 | I thought there was too much inconsistency in the AICaries app. |  |  |  |  |  |  |
|  | *Scoring algorism (point)* | 4 | 3 | 2 | 1 | 0 |  |
| 7 | I would imagine that most people would learn to use the AICaries app very quickly. |  |  |  |  |  |  |
|  | *Scoring algorism (point)* | 0 | 1 | 2 | 3 | 4 |  |
| 8 | I found the AICaries app very awkward to use. |  |  |  |  |  |  |
|  | *Scoring algorism (point)* | 4 | 3 | 2 | 1 | 0 |  |
| 9 | I felt very confident using AICaries app. |  |  |  |  |  |  |
|  | *Scoring algorism (point)* | 0 | 1 | 2 | 3 | 4 |  |
| 10 | I needed to learn a lot of things before I could get going with the AICaries app. |  |  |  |  |  |  |
|  | *Scoring algorism (point)* | 4 | 3 | 2 | 1 | 0 |  |

***Total score = sum of all 10 items * 2.5**

**Appendix 3: AICaries Step 1 usability test - Patient Taking Photos Qualitative Assessment Sheet**

**(Study Team Use)**

Notes:

- In the Usability test, patients will take a series of intraoral photos for their child. The session will be recorded.
- The video assessment will be conducted by research assistant (a dentist) trained by study investigators.

| **Tasks by the patients** | **Time-spent (minute)** | **Challenges** | | | |
| --- | --- | --- | --- | --- | --- |
|  |  | **No challenges** | **Cosmetic (minor)** | **Moderate** | **Critical** |
| - Locate AICaries app on the study smartphone |  |  |  |  |  |
| - Navigation of the app interface |  |  |  |  |  |
| - Accessing and completing the ADA risk assessment |  |  |  |  |  |
| - Taking diagnostic photographs of their child’s front teeth *using AICaries* |  |  |  |  |  |
| - Taking diagnostic photographs of their child’s posterior teeth *using AICaries* |  |  |  |  |  |
| - Connecting intraoral cameras with tablet |  |  |  |  |  |
| - Use intraoral camera take photos for their children (front teeth) |  |  |  |  |  |
| - Use intraoral camera take photos for their children (back teeth) |  |  |  |  |  |
| - Others |  |  |  |  |  |

Note:

*Cosmetic*: minor, took longer than expected, but able to resolve without help

*Moderate:* major delay and/or frustration, required verbal guidance by the research assistant

*Critical*: requiring assistance to proceed or demo of the research assistant

**Appendix 4: AICaries Study Intraoral Photos Assessment Sheet**

**(Study Team Use)**

Note: The quantity and quality of intraoral photos will be assessed by a study research assistant (a dentist) trained by study investigators.

| Location | Total | Clear images | Diagnostic images |
| --- | --- | --- | --- |
| Number of total intraoral photos |  |  |  |
| Break down | | | |
| Front view |  |  |  |
| Buccal-Upper molars (left) |  |  |  |
| Buccal-Upper molars (right) |  |  |  |
| Lingual-Upper molars (left) |  |  |  |
| Lingual-Upper molars (right) |  |  |  |
| Lingual- Upper premolars (left) |  |  |  |
| Lingual- Upper premolars (right) |  |  |  |
| Lingual-Upper front |  |  |  |
| Lingual-Lower molars (left) |  |  |  |
| Lingual-Lower molars (right) |  |  |  |
| Lingual-Lower premolars (left) |  |  |  |
| Lingual-Lower premolars (right) |  |  |  |
| Lingual-Lower front |  |  |  |
| Occlusal-Upper molars (left) |  |  |  |
| Occlusal-Upper molars (right) |  |  |  |
| Occlusal- Upper premolars (left) |  |  |  |
| Occlusal- Upper premolars (right) |  |  |  |
| Occlusal-Upper front |  |  |  |
| Occlusal-Lower molars (left) |  |  |  |
| Occlusal-Lower molars (right) |  |  |  |
| Occlusal-Lower premolars (left) |  |  |  |
| Occlusal-Lower premolars (right) |  |  |  |
| Occlusal-Lower front |  |  |  |

**Appendix 5: AICaries step 2 Field Testing Interview guide**

**(Semi-structured)**

**Notes:**

- This is a semi-structured interview guide designed to be conducted with patients in a 30-min interview setting.
- This interview includes questions to assess user’s perception of using AICaries app and taking intraoral photos for young children.
- The phone interview will be conducted by research assistant (a dentist) trained by study investigators who have expertise in mhealth and qualitative research.

***Date: _____/_____/_____ (mm/dd/yy)***

***Interviewer: _____________________***

***Study Participant number: __________***

***Introduction (script)***

*“Hello, my name is ______. I am with the AICaries study group and I would like to talk with you about your experience of using AICaries smartphone app.”*

*“Thank you for agreeing to speak to me! This interview will take approximately 30 minutes and will be recorded.”*

*“What we talked about in this interview will not be released to personnel outside of our study team”*

*“Your participation is completely voluntary. If you do not wish to answer any question you do not have to do so. You are free to end the conversation at any time”*

*“Before I turn on the tape recorder do you have any questions?”*

*“Now I am going to turn on the tape recorder, is that ok?”*

**Perception of using AICaries**

1. Please tell me about your overall experience of using AICaries smartphone app?
2. Please tell me about challenges or frustrations you encountered while using AICaries smartphone app?

*Prompts – What kind of challenges / frustrations*

*Did you resolve it?*

*How did you resolve it?*

*Anything else (until no more)?*

1. What are the benefits, if any, of using AICaries smartphone app?

*Prompts – Convenience?*

*Maintain or improve oral health*

*Any other benefits (until no more)?*

1. What suggestions do you have to improve the smartphone app?

*Prompts – What* else would you wish to include in the AICaries smartphone app?

*What would make it easier to use?*

1. How likely would you recommend your family members and friends to use AICaries smartphone app?

*Prompts – Not likely, neutral, likely*

**Taking teeth photos**

1. Please tell me your experience in taking pictures of your child’s teeth?

2. What problems did you run into?

*Prompt – Any problems during using AICaries app camera module?*

*Other problems (until no more)?*

3. How did you resolve these problems?

4. What did you like best about the app? What did you like least about the app?

5. What suggestions do you have for making it easier for taking pictures of children’s teeth?

**Conclusion**

*“What other things you would like us to know about your experience or thoughts about AICaries smartphone app?”*

“Thank you!”
