# Supplementary material for: Assessing a Smartphone App (AICaries) That Uses Artificial Intelligence to Detect Dental Caries in Children and Provides Interactive Oral Health Education: Protocol for a Design and Usability Testing Study
Source: JMIR Res Protoc. 2021 Oct 22;10(10):e32921. doi: 10.2196/32921 (PMC8571694; doi:10.2196/32921)
Supplement: Multimedia Appendix 2 [file resprot_v10i10e32921_app2.pdf]

**1R21DE030251-01 Xiao, Jin**

**RESUME AND SUMMARY OF DISCUSSION:** The application proposes to develop and test a smartphone app that detects early childhood caries. It tackles a highly significant problem of lack of timely dental caries diagnosis, and if successful, the technology can potentially reduce caries incidences. The multidisciplinary investigative team includes expertise in artificial intelligence, pediatric dentistry, disparities, and implementation science. The approach is well developed and supported by strong preliminary data. The caries detection module will allow detection from different angles, and plans for validation against detection from trained individuals and evaluation of usability are strong. The community infrastructure is in place to support the testing of usability and acceptability. Some minor concerns are noted, such as the focus on front teeth only and impacts limited to those with smartphones only. Overall, enthusiasm is high for this significant and innovative application, and the project is viewed to have a high impact on caries detection to reduce dental health disparities.

**DESCRIPTION (provided by applicant):** Early childhood caries (ECC) is the most common chronic childhood disease, with nearly 1.8 billion new cases per year globally. ECC afflicts approximately 55% of low-income and minority US preschool children, resulting in harmful short- and long-term effects on health and quality of life. The current biomedical approach to control the ECC pandemic has had limited success. It primarily focuses on restorative procedures rather than population-wide preventive strategies. Clinical evidence shows that caries is reversible if detected and addressed in its early stages. However, many low-income US children often have poor access to pediatric dental services. In this underserved group, dental caries is often diagnosed at a late stage when extensive restorative treatment is needed. We believe that with more than 85% of lower-income Americans owning a smartphone, mHealth tools hold great promise to achieve patient-driven early detection and risk control of ECC. Our long-term goal is to develop strategies that use mHealth tools to achieve early detection and prevention of ECC at a broad population base. Our previous innovative work has led to a novel prototype of an artificial intelligence (AI) -powered smartphone app, AICaries, to be used by children's parents/caregivers. This AICaries app prototype offers a) AI-powered caries detection using photos of children's teeth taken by the parents' smartphones, b) interactive caries risk assessment, and c) personalized education on reducing children's ECC risk. The preliminary AI- powered caries detection module demonstrated a satisfactory sensitivity and specificity for front teeth caries detection, using 6,895 annotated tooth images from 1,277 photos. We have recently built an archive of > 100,000 high-quality intra-oral photos that is ready to be used for finalizing the development of a reliable automatic detection algorithm. The immediate objectives of the study are - AIM 1: complete the development of AICaries smartphone app, maximize its caries detection performance, and achieve a caries detection sensitivity and specificity that are comparable to trained dental practitioners; AIM 2: employ a community-based participatory research strategy to conduct moderated testing and refinement of the app usability, and non-moderated field testing of the app feasibility/acceptability. Our multidisciplinary team is well-positioned for proposal success with needed expertise in computer science, AI imaging recognition, oral health care, mHealth, disparity research, patient education and community engagement. The AICaries app could facilitate early detection of ECC for many underserved US children, who often have poor access to pediatric dental services. Using AICaries, parents can use their regular smartphones to take photo of their children’s teeth and detect ECC aided by AICaries, so that they can actively seek treatment for their children at an early and reversible stage of ECC. Using AICaries, parents can also obtain essential knowledge on reducing their children's caries risk. Data from this R21 will support a R01 clinical trial that evaluates the real-world impact of using this innovative smartphone app on early detection and prevention of ECC among low-income children.

**PUBLIC HEALTH RELEVANCE:** Although largely preventable, early childhood caries (ECC) remains the most common chronic childhood disease, disproportionately afflicts vulnerable parts of the

population and has a substantial adverse impact on children, families, and healthcare systems. Our multidisciplinary team is proposing to use an Artificial Intelligence-powered mHealth tool coupled with a community engagement strategy to revolutionize the detection and monitoring of ECC at the patient level, which may pave the way for improving oral heath among low-income children.

**CRITIQUE 1**

Significance: 2
Investigator(s): 2
Innovation: 1
Approach: 2
Environment: 2

**Overall Impact:** This application proposes to develop and evaluate an artificial intelligence supported smartphone app to detect early childhood caries among children less than 5 years from images taken by parents. This study is innovative and significant even though it is not clear to what extent such an app would be useful to improve caries detection and prevention among the lower-income Americans who may not have a smartphone, sufficient Internet bandwidth and computer skills literacy. Regardless of this drawback, the outcome of this application can transform the delivery of dental care by empowering people to detect potential oral health problems and undertake appropriate preventive management. Such an approach could have the potential to reduce dental care costs and enhance the oral health of US population through improvement in oral health literacy and prevention.

**1. Significance:**

**Strengths**

- The proposal has the potential to educate parents of young children regarding early childhood caries detection and management.

**Weaknesses**

- It is uncertain to what extent this app would be used by the parents of children in the underserved population. According to recent pew research center reports digital divide persists between lower and higher income Americans. Moreover, in addition to owning smartphone, other factors such as computer literacy and skills, and having access to Internet influence adoption and use of smartphone apps.

**2. Investigator(s):**

**Strengths**

- Excellent multidisciplinary team with expertise in pediatric dentistry, artificial intelligence and image recognition, implementation research, mHealth, patient oral education and community engagement.

**Weaknesses**

- None noted

**3. Innovation:**

**Strengths**

- Early detection of caries is detected mostly by visual assessment and retrieving that information from digital images is innovative.

**Weaknesses**

- None noted

**4. Approach:**

**Strengths**

- Strong preliminary results demonstrating the feasibility of adoption and use of the app by the target population; already developed shell for the app, availability of a corpus of 100,000 anterior teeth images to jumpstart the development of the algorithm and the development of an annotation software to automate annotation of images. All these results enhance the confidence of the success of the application.
- Well-developed approach covering all aspects of development and evaluation that includes, developing the caries detection module, intraoral image acquisition, automatic tooth/teeth localization and detection from different angles based on context and caries status assessment. The AI-powered caries detection module will be evaluated against trained experts. The apply will be finalized through iterative usability testing and refinement using think-aloud protocols and finally, will be tested in the field for usability and acceptability.

**Weaknesses**

- Not sure why posterior teeth and lower anterior teeth are excluded.
- It is not clear to what extent the large repository of images is representative of the images taken by the target user population of the app. If the model is trained based on images taken by dental professionals and dental students, it may affect the accuracy when a person from an underserved community takes the image.

**5. Environment:**

**Strengths**

- Eastman Dental Institute and University of Rochester Medical Center are well equipped to support this study.

**Weaknesses**

- None

**Protections for Human Subjects:**

Acceptable Risks and/or Adequate Protections

- Acceptable

Data and Safety Monitoring Plan (Applicable for Clinical Trials Only):

Not Applicable (No Clinical Trials)

**Inclusion Plans:**

- Sex/Gender: Distribution justified scientifically
- Race/Ethnicity: Distribution justified scientifically
- Inclusion/Exclusion Based on Age: Distribution justified scientifically

**Vertebrate Animals:**
Not Applicable (No Vertebrate Animals)

**Biohazards:**
Not Applicable (No Biohazards)

**Resource Sharing Plans:**
Acceptable

**Authentication of Key Biological and/or Chemical Resources:**
Acceptable

**Budget and Period of Support:**
Recommend as Requested

**CRITIQUE 2**

Significance: 1
Investigator(s): 2
Innovation: 1
Approach: 1
Environment: 1

**Overall Impact:** Although largely preventable, early childhood caries (ECC) remains the most common chronic childhood disease, disproportionately afflicts vulnerable parts of the population and has a substantial adverse impact on children, families, and healthcare systems. This is an innovative and highly significant proposal that builds on previous work and prototype to use an Artificial Intelligence-powered mHealth tool coupled with a community engagement strategy to revolutionize the detection and monitoring of early ECC at the patient level, with the potential to pave the way for improving oral heath among low-income children. The multidisciplinary team is well-positioned for proposal success with the needed expertise (computer science, biostatistics, AI imaging recognition, oral health care, mHealth, disparity research, patient education, and community engagement), funded studies and publications that support this proposal. Although there are 3 PIs on this proposal the Multi-PI Plan delineates, roles, responsibilities, and lines of communication. A weakness is the exclusion of a Biographical Sketch for Co-I, Dr. Oriana Ly-Mapes, to verify her qualifications and expertise in patient education to modifying the smartphone app oral health education content and serve as one of the gold standard dental examiners for AICaries detection module. The environment is well suited for this proposal. The approach is rigorous and robust and includes two aims: 1) Complete the development of AICaries smartphone app, maximize its caries detection performance, and achieve a caries detection

sensitivity and specificity that are comparable to trained dental practitioners; and 2) Employ a community-based participatory research strategy to conduct: a) iterative moderated usability testing and app refinement using Think-aloud and Instant Data Analysis strategies moderated testing; and b) unmoderated field testing for app feasibility and acceptability by assessing the number/quality of teeth images taken by the parents for their children and parents' satisfaction. The enrollment plan for both aims of the study include a justification for the predominately female sex of the parents, and children < 5 years. Ethnicity/minority are appropriately represented. I am highly enthusiastic for this excellent proposal.

**1. Significance:**

**Strengths**

- The current biomedical approach to control the ECC pandemic primarily focuses on restorative procedures rather than population-wide preventive strategies and has had limited success.
- With more than 85% of lower-income Americans owning a smartphone, mHealth tools hold great promise to achieve patient-driven early detection and risk control of ECC.
- The AICaries app shows the potential to facilitate early detection of ECC for many underserved US children, who often have poor access to pediatric dental services.
- Data from this R21 will support a R01 clinical trial that evaluates the real-world impact of using this innovative smartphone app on early detection and prevention of ECC among low-income children.

**Weaknesses**

- None noted.

**2. Investigator(s):**

**Strengths**

- The exceptional multidisciplinary team is well-positioned for proposal success with needed expertise in computer science, biostatistics, AI imaging recognition, oral health care, mHealth, disparity research, patient education, and community engagement. The investigators have a strong history of funded studies and publications that support this proposal.
- The team includes 3 PIs, 3 Co-Is, and 1 consultant. The Multi-PI Leadership defines the roles, responsibilities, communication structure and conflict resolution plan.

**Weaknesses**

- The Profile - Senior/Key Person nor a Biographical Sketch is included for Co-I Dr. Oriana Ly-Mapes, who will provide her expertise in patient education to modifying the smartphone app oral health education content and serve as one of the gold standard dental examiners for AICaries detection module sensitivity/specificity testing. Her qualifications for this Co-I work need to be verified.

**3. Innovation:**

**Strengths**

- Integrating the mobile health concept into dentistry to achieve population-wide caries prevention is extremely innovative and offers a vehicle to promote early intervention.

- This proposal is based on previous innovative work that developed a novel prototype of an artificial intelligence (AI) -powered smartphone app, AICaries, to be used by children's parents/caregivers.
- Using AICaries, parents can use their regular smartphones to take photos of their children's teeth and detect ECC aided by AICaries, so that they can actively seek treatment for their children at an early and reversible stage.

**Weaknesses**

- None noted.

**4. Approach:**

**Strengths**

- The preliminary work on the AICaries app prototype offers a) AI-powered caries detection using photos of children's teeth taken by the parents' smartphones, b) interactive caries risk assessment, and c) personalized education on reducing children's ECC risk. The preliminary AI-powered caries detection module demonstrated a satisfactory sensitivity and specificity for front teeth caries detection.
- This proposal builds on this prototype with two rigorous and robust aims to:
  - Complete the development of AICaries smartphone app, maximize its caries detection performance, and achieve a caries detection sensitivity and specificity that are comparable to trained dental practitioners. This aim uses a recently built archive of > 100,000 high-quality intra-oral photos that is ready to be used for finalizing the development of a reliable automatic detection algorithm. This aim includes the development of three sub-modules: a) an IntraOral Photo Acquisition module that guides the novice users through the photo-taking process for high image quality; b) a Tooth Identification and Localization module that automatically identifies each tooth from photos taken by the users; and c) a Caries Status Assessment module that assigns a severity score to each identified tooth and generates reports on caries status. This aim includes sensitivity and specificity testing on caries detection.
- Employ a community-based participatory research strategy to conduct a) iterative moderated usability testing and app refinement using Think-aloud and Instant Data Analysis strategies moderated testing; and b) unmoderated field testing for app feasibility and acceptability by assessing the number/quality of teeth images taken by the parents for their children and parents' satisfaction.
- There is a well-designed plan with alternative approaches to handle anticipated challenges.
- The enrollment plan for both aims of the study include a justification for the predominately female sex of the parents, and children < 5 years. Ethnicity/minority are appropriately represented. The Recruitment and Retention Plan provides appropriate strategies for retaining the participants throughout the 2 studies.
- Study 1 plans to enroll 10 pairs of parents and their children, a total of 20 participants to test the usability of AICaries.
- Study 2 plans to enroll 32 pairs of parents and their young children, a total of 64 participants to field test AICaries.

**Weaknesses**

- None noted.

**5. Environment:**

**Strengths**

- The resources and facilities at the University of Rochester including their Eastman Institute for Oral Health and clinics provide an excellent environment for this study.
- Community partners include the Healthy Baby Network, with their Executive Director serving as a consultant on this proposal, and the Monroe county Nurse-Family Partnership (NFP).
- The study uses the REDCap Consortium, disseminated by Vanderbilt, for electronic collection and management of research and clinical trial data.

**Weaknesses**

- None noted.

**Study Timeline:**

**Strengths**

- This is not a clinical trial. The timeline seems appropriate for the two aims of this study.

**Weaknesses**

- None noted by reviewer.

**Protections for Human Subjects:**

Acceptable Risks and/or Adequate Protections

- Study 1 and Study 2 have a well-designed plan for the Protection of Human Subjects.

Data and Safety Monitoring Plan (Applicable for Clinical Trials Only):

Not Applicable (No Clinical Trials)

**Inclusion Plans:**

- Sex/Gender: Distribution justified scientifically
- Race/Ethnicity: Distribution justified scientifically
- Inclusion/Exclusion Based on Age: Distribution justified scientifically
- Study 1 plans to enroll 10 pairs of parents and their children, a total of 20 participants to test the usability of AICarries.
- Study 2 plans to enroll 32 pairs of parents and their young children, a total of 64 participants to field test AICarries.
- Both study samples include primarily economically and socially disadvantaged parents (mainly mothers) and their young children (< 5 years of age). Investigators expect the participants to be 40% White, 45% Black or African American, 5% Asian and 10% other race; and the composition of ethnical groups among the study sample is 80% non-Hispanic and 20% Hispanic. Since mothers are the primary caregiver for children, the ratio between female and male of the parent participants is expected to be 4:1, with 80% mothers and 20% fathers.

**Vertebrate Animals:**

Not Applicable (No Vertebrate Animals)

**Biohazards:**

Not Applicable (No Biohazards)

**Resource Sharing Plans:**

Unacceptable

- The Resource Sharing Plan only includes presentations at local institution's communities and national and international scientific meetings and publication in journals.
- The sharing of de-identified data is not discussed.

**Budget and Period of Support:**

Recommend as Requested

**CRITIQUE 3**

Significance: 2
Investigator(s): 1
Innovation: 2
Approach: 3
Environment: 1

**Overall Impact:** The project addresses the lack of access to early childhood caries (ECC) diagnosis and treatment. The proposal has two main aims: (1) develop a smartphone tool for detecting caries; (2) test usability of the tool. The proposal is generally rigorous and will likely be completed successfully. However, there are a few questions that the proposal does not address: (1) How accurately can the smartphone tool detect early stage ECC? Early detection is mentioned as the key to transforming treatment from “individual-level restorative procedures” to “population-wide preventive strategies”. (2) How accurately does the tool detect ECC from photos taken on a smartphone? (3) What are the minimum quality criteria of the images needed to achieve adequate classification accuracy? Without the answer to (1), the impact of the proposal is diminished. Without the answers to (2) and (3), it will be challenging to justify a clinical trial in the future.

**1. Significance:**

**Strengths**

- The project addresses the following problem in the field of oral health: control of early childhood caries (ECC) is hindered by patients’ lack of access to early disease detection.
- Rigor of prior research: previous work on technology infrastructure, image database creation and user research all support the feasibility of the proposed work.

**Weaknesses**

- Rigor of prior research: The initial AI caries tool has a relatively low sensitivity. In addition, it is unclear how classification accuracy changes for different levels of caries severity. If accuracy is worse for early stage caries, then its utility as an effective preventive tool is diminished.
- The proposal does not address how to ensure caregivers' adherence to a preventative health regimen. Without adherence, the proposed technology will have a limited impact on oral healthcare.

**2. Investigator(s):**

**Strengths**

- The investigators have complementary and relevant expertise, from imaging to child oral health and tele dentistry.
- Multi-PI plan satisfactorily addresses leadership approach, governance and organizational structure.

**Weaknesses**

- None

**3. Innovation:**

**Strengths**

- Application seeks to shift clinical practice to a more preventative approach with the help of a novel smartphone-based instrument.

**Weaknesses**

- The concept of smartphone-based teledentistry (e.g. oral cancer screening) is not particularly novel. (Minor)

**4. Approach:**

**Strengths**

- Aim 1: General app development strategy is adequate.
- Aim 1: Evaluation plan includes explicit quantitative targets for caries detection accuracy.
- Aim 2: Generally adequate strategy for usability testing and iterative design.
- Aim 2: Adequate protection of human subjects from risk to confidentiality.
- Aim 2: Inclusion of children < 5 justified.

**Weaknesses**

- Aim 1: Addressing weaknesses in prior research: No plans provided to evaluate classifier accuracy for early stage caries.
- Aim 1: No plans proposed to evaluate caries level classification accuracy.
- Aim 1: No plans proposed to test the classifier on smartphone images.
- Aim 1: Sex as a biological variable: not addressed.
- Aim 2: Evaluation plan does not identify explicit quantitative targets for ease-of-use, ease-of-learning, etc.

- Aim 2: Requiring users to watch an instructional video before use can pose a significant barrier to acceptability of the tool. An alternative strategy would be to make the tool more intuitive and self-explanatory, using information gleaned during the usability testing phase.
- Aim 2: Sex as a biological variable: not addressed.

**5. Environment:**

**Strengths**

- Project will benefit from patient population access via Eastman Perinatal Dental Clinic, CBPR sites and the Healthy Baby Network.
- Project will benefit from involvement in the Patient-Empowered Advisory Committee (PEAC) and from access to intraoral photo database.

**Weaknesses**

- None

**Protections for Human Subjects:**

Acceptable Risks and/or Adequate Protections

- Adequate protection of human subjects from risk to confidentiality.

Data and Safety Monitoring Plan (Applicable for Clinical Trials Only):

Not Applicable (No Clinical Trials)

**Inclusion Plans:**

- Sex/Gender: Distribution justified scientifically
- Race/Ethnicity: Distribution justified scientifically
- Inclusion/Exclusion Based on Age: Distribution not justified scientifically
- Exclusion of parents > 50 years not justified scientifically. Also, grandparents are often primary caregivers of children in low income households.

**Vertebrate Animals:**

Not Applicable (No Vertebrate Animals)

**Biohazards:**

Not Applicable (No Biohazards)

**Resource Sharing Plans:**

- Adequate plan to share research findings at conferences and in publications.

**Authentication of Key Biological and/or Chemical Resources:**

Not Applicable (No Relevant Resources)

**Budget and Period of Support:**
Recommend as Requested

**THE FOLLOWING SECTIONS WERE PREPARED BY THE SCIENTIFIC REVIEW OFFICER TO SUMMARIZE THE OUTCOME OF DISCUSSIONS OF THE REVIEW COMMITTEE, OR REVIEWERS' WRITTEN CRITIQUES, ON THE FOLLOWING ISSUES:**

**PROTECTION OF HUMAN SUBJECTS: ACCEPTABLE**

**INCLUSION OF WOMEN PLAN: ACCEPTABLE**

**INCLUSION OF MINORITIES PLAN: ACCEPTABLE**

**INCLUSION ACROSS THE LIFESPAN: ACCEPTABLE**

**COMMITTEE BUDGET RECOMMENDATIONS: The budget was recommended as requested.**

---

Footnotes for 1 R21 DE030251-01; PI Name: Xiao, Jin

NIH has modified its policy regarding the receipt of resubmissions (amended applications).See Guide Notice NOT-OD-18-197 at https://grants.nih.gov/grants/guide/notice-files/NOT-OD-18-197.html. The impact/priority score is calculated after discussion of an application by averaging the overall scores (1-9) given by all voting reviewers on the committee and multiplying by 10. The criterion scores are submitted prior to the meeting by the individual reviewers assigned to an application, and are not discussed specifically at the review meeting or calculated into the overall impact score. Some applications also receive a percentile ranking. For details on the review process, see http://grants.nih.gov/grants/peer_review_process.htm#scoring.

**MEETING ROSTER**

**Center for Scientific Review Special Emphasis Panel**
**CENTER FOR SCIENTIFIC REVIEW**
**Clinical Informatics and Digital Health**

**ZRG1 BCHI-E (09)**
**06/25/2020**

**Notice of NIH Policy to All Applicants:** Meeting rosters are provided for information purposes only. Applicant investigators and institutional officials must not communicate directly with study section members about an application before or after the review. Failure to observe this policy will create a serious breach of integrity in the peer review process, and may lead to actions outlined in NOT-OD-14-073 at https://grants.nih.gov/grants/guide/notice-files/NOT-OD-14-073.html and NOT-OD-15-106 at https://grants.nih.gov/grants/guide/notice-files/NOT-OD-15-106.html, including removal of the application from immediate review.

## CHAIRPERSON(S)

JAFARI, ROOZBEH, PHD
ASSOCIATE PROFESSOR
DEPARTMENTS OF BIOMEDICAL ENGINEERING,
COMPUTER SCIENCE AND ENGINEERING,
AND ELECTRICAL AND COMPUTER ENGINEERING
TEXAS A & M UNIVERSITY
COLLEGE STATION, TX 77843

## MEMBERS

AFSHAR, MAJID, MD
ASSISTANT PROFESSOR
DEPARTMENT OF MEDICINE
DEPARTMENT OF HEALTH INFORMATICS
AND DATA SCIENCE
LOYOLA UNIVERSITY CHICAGO
MAYWOOD, IL 60153

AHAMED, SHEIKH IQBAL, PHD
PROFESSOR
DEPARTMENT OF MATHEMATICS, STATISTICS
AND COMPUTER SCIENCE
MARQUETTE UNIVERSITY
MILWAUKEE, WI 53233

ALPERN, ELIZABETH RACHEL, MD
PROFESSOR
DEPARTMENT OF PEDIATRICS
CENTER FOR HEALTHCARE STUDIES
FEINBERG SCHOOL OF MEDICINE
NORTHWESTERN UNIVERSITY
CHICAGO, IL 60611

AUDU, MUSA L, PHD
PROFESSOR
DEPARTMENT OF BIOMEDICAL ENGINEERING
ADVANCED PLATFORM TECHNOLOGY CENTER
CASE WESTERN RESERVE UNIVERSITY
CLEVELAND, OH 44106

CARREIRO, STEPHANIE P, MD
ASSISTANT PROFESSOR
DIVISION OF TOXICOLOGY
DEPARTMENT OF EMERGENCY MEDICINE
UNIVERSITY OF MASSACHUSETTS MEDICAL SCHOOL
WORCESTER, MA 01655

CHON, KI H, PHD
PROFESSOR
BIOMEDICAL ENGINEERING
UNIVERSITY OF CONNECTICUT
STORRS, CT 06269

CULLEY, JOAN MARIE, MPH, PHD
PROFESSOR
COLLEGE OF NURSING
UNIVERSITY OF SOUTH CAROLINA
COLUMBIA, SC 29208

DEXHEIMER, JUDITH W, PHD
ASSOCIATE PROFESSOR
DEPARTMENT OF PEDIATRICS AND BIOMEDICAL
INFORMATICS, DIVISION OF EMERGENCY MEDICINE
CINCINNATI CHILDREN'S HOSPITAL MEDICAL CENTER
UNIVERSITY OF CINNCINNATI
CINCINANTI, OH 45229

DUNCAN, DOMINIQUE, PHD
ASSISTANT PROFESSOR
LABORATORY OF NEURO IMAGING
USC STEVENS NEUROIMAGING AND INFORMATICS
INSTITUTE
KECK SCHOOL OF MEDICINE
UNIVERSITY OF SOUTHERN CALIFORNIA
LOS ANGELES, CA 90033

ESWARAN, HARI, PHD
PROFESSOR
DEPARTMENT OF OBSTETRICS AND GYNECOLOGY
UNIVERSITY OF ARKANSAS FOR MEDICAL SCIENCES
LITTLE ROCK, AR 72205

ODAME, KOFI, PHD
ASSOCIATE PROFESSOR
DEPARTMENT OF ENGINEERING SCIENCES
THAYER COLLEGE OF ENGINEERING
DARTMOUTH COLLEGE
HANOVER, NH 03755

SANO, AKANE, PHD
ASSISTANT PROFESSOR
DEPARTMENT OF ELECTRICAL COMPUTER ENGINEERING
AND COMPUTER SCIENCE
RICE UNIVERSITY
HOUSTON, TX 77251

SHAW, RYAN JEFFREY, PHD
ASSOCIATE PROFESSOR
SCHOOL OF NURSING
CENTER FOR HEALTH INFORMATICS
CENTER FOR PRECISION MEDICINE
DUKE UNIVERSITY
DURHAM, NC 27708

SUBBIAN, VIGNESH, PHD
ASSISTANT PROFESSOR
DEPARTMENT OF BIOMEDICAL ENGINEERING
DEPARTMENT OF SYSTEMS AND INDUSTRIAL ENGINEERING
COLLEGE OF ENGINEERING
THE UNIVERSITY OF ARIZONA
TUCSON, AZ 85721

THYVALIKAKATH, THANKAM P, DMD, PHD
ASSOCIATE PROFESSOR
DEPARTMENT OF CARIOLOGY, OPERATIVE DENTISTRY
AND DENTAL PUBLIC HEALTH
SCHOOL OF DENTISTRY
INDIANA UNIVERSITY
INDIANAPOLIS, IN 46202

XU, WENYAO, PHD
ASSOCIATE PROFESSOR
DEPARTMENT OF COMPUTER SCIENCE AND ENGINEERING
UNIVERSITY AT BUFFALO
STATE UNIVERSITY OF NEW YORK
BUFFALO, NY 14260

ZHANG, GQ, PHD
VICE PRESIDENT AND CHIEF DATA SCIENTIST
PROFESSOR OF MEDICINE, BIOMEDICAL INFORMATICS AND
PUBLIC HEALTH
UNIVERSITY OF TEXAS HEALTH SCIENCE CENTER
HOUSTON, TX 77030

ZHOU, LI, PHD
ASSOCIATE PROFESSOR
DEPARTMENT OF MEDICINE
BRIGHAM AND WOMEN'S HOSPITAL
HARVARD MEDICAL SCHOOL
SOMERVILLE, MA 02145

## SCIENTIFIC REVIEW OFFICER

LIANG, WENCHI, PHD
SCIENTIFIC REVIEW OFFICER
CENTER FOR SCIENTIFIC REVIEW
NATIONAL INSTITUTES OF HEALTH
BETHESDA, MD 20892

VEERARAGHAVAN, SUDHA, PHD
SCIENTIFIC REVIEW OFFICER
CENTER FOR SCIENTIFIC REVIEW
NATIONAL INSTITUTES OF HEALTH
BETHESDA, MD 20892

## EXTRAMURAL SUPPORT ASSISTANT

JONES, BELINDA
EXTRAMURAL SUPPORT ASSISTANT
CENTER FOR SCIENTIFIC REVIEW
NATIONAL INSTIUTIES OF HEALTH
BETHESDA, MD 20892

Consultants are required to absent themselves from the room during the review of any application if their presence would constitute or appear to constitute a conflict of interest.
